# Wetting Behavior of A*-block-*(B-*random*-C) Copolymers with Equal Block Surface Energies on Surfaces Functionalized with B-*random*-C Copolymers

**DOI:** 10.1021/acs.langmuir.3c02065

**Published:** 2023-10-02

**Authors:** Hongbo Feng, Benjamin Kash, Soonmin Yim, Kushal Bagchi, Gordon S. W. Craig, Wen Chen, Stuart J. Rowan, Paul F. Nealey

**Affiliations:** † Pritzker School of Molecular Engineering, University of Chicago, 5640 S. Ellis Avenue, Chicago, Illinois 60637, United States; ‡ Department of Chemistry, University of Chicago, 5735 S. Ellis Avenue, Chicago, Illinois 60637, United States; § Chemical Sciences and Engineering Division, Argonne National Laboratory, 9700 S. Cass Avenue, Lemont, Illinois 60439, United States; ∥ Center for Molecular Engineering, Materials Science Division, Argonne National Laboratory, 9700 S. Cass Avenue, Lemont, Illinois 60439, United States

## Abstract

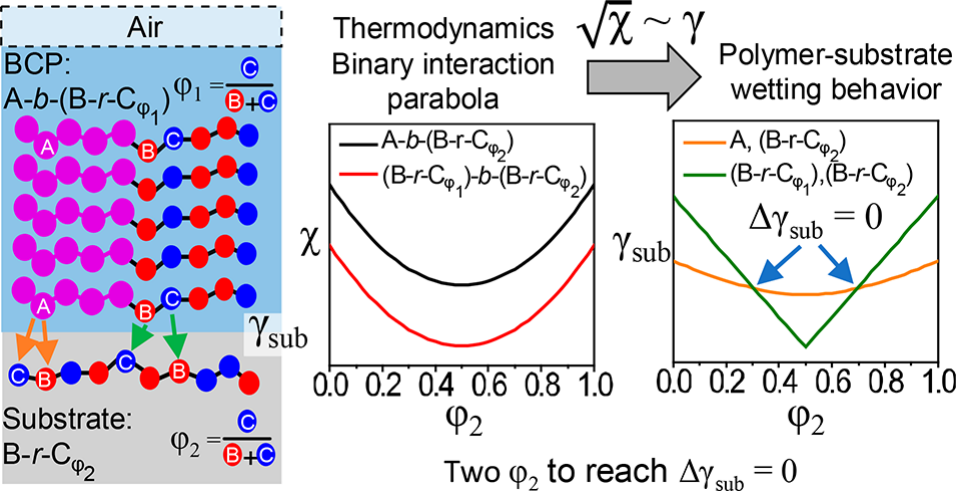

To form nanopatterns with self-assembled block copolymers (BCPs), it is desirable to have through-film domains that are oriented perpendicular to the substrate. The domain orientation is determined by the interfacial interactions of the BCP domains with the substrate and with the free surface. Here, we use thin films of two different sets of BCPs with A*-block-*(B-*random*-C) architecture matched with a corresponding B-*random*-C copolymer nanocoating on the substrate to demonstrate two distinct wetting behaviors. The two sets of A*-b-*(B-*r*-C) BCPs are made by using thiol–epoxy click chemistry to functionalize polystyrene*-block-*poly(glycidyl methacrylate) with trifluoroethanethiol (TFET) and either 2-mercaptopyridine (2MP) or methyl thioglycolate (MTG). For each set of BCPs, the composition ratio of the two thiols in the BCP (φ_1_) is found that results in the two blocks of the modified BCP having equal surface energies (Δγ_air_ = 0). The corresponding B-*r*-C random copolymers were synthesized and used to modify the substrate, and the composition ratio (φ_2_) values that resulted in the two blocks of the BCP having equal interfacial energy with the substrate (Δγ_sub_ = 0) were determined with scanning electron microscopy. The correlation between each block’s γ_sub_ value and the interaction parameter, χ, is employed to explain the different wetting behaviors of the two sets of BCPs. For the thiol pair 2MP and TFET, the values of φ_1_ and φ_2_ that lead to Δγ_air_ = 0 and Δγ_sub_ = 0, respectively, are significantly different. A similar difference was observed between the φ_1_ and φ_2_ values that lead to Δγ_air_ = 0 and Δγ_sub_ = 0 for the BCPs made with the thiol pair MTG and TFET. In the latter case, for Δγ_sub_ = 0 two windows of φ_2_ are identified, which can be explained by the thermodynamic interactions of the specific thiol pair and the A-*b*-(B-*r*-C) architecture.

## Introduction

The self-assembly of block copolymers (BCPs) offers a unique pathway toward a plethora of nanostructures, governed by the Flory–Huggins interaction parameter (χ), degree of polymerization (*N*), and volume fraction of blocks (*f*).^1^ Considerable research efforts have been made to utilize these structures for a broad range of applications including directed self-assembly (DSA) for lithography and precursors of nonporous materials.^2,3^ The thermodynamics of DSA of thin films of BCPs, which is being developed for semiconductor manufacturing^4,5^ and has potential applications in high density magnetic recording^6^ and a host of other non-semiconductor applications,^7^ is impacted significantly by interfacial interactions at the free surface, between BCP domains, and between the BCP and the patterned polymer on the substrate.^8^ For most applications, perpendicularly oriented through-film domains that form via thermal annealing are necessary. For self-assembly without a chemically patterned substrate, perpendicular domain orientation requires that there is no difference between the BCP domains in terms of their interfacial energy (γ) with air (Δγ_air_ = 0) or with the polymer coating on the substrate (Δγ_sub_ = 0).^9^ However, with the growing interest in developing BCPs able to form features with small dimensions, which require a large χ, it is often challenging to achieve Δγ_air_ = 0 because BCPs with high χ typically have constituent blocks with large differences in polarity and therefore different γ_air_. For example, polydimethylsiloxane (PDMS) domains will segregate at the free surface when annealing polystyrene*-block-*polydimethylsiloxane thin films on a silicon substrate because of the much lower surface energy of the PDMS domain.^10^ A number of approaches have been reported to access perpendicular orientation including solvent vapor annealing,^11−13^ topcoats,^14−16^ and external force fields.^10,17,18^ These approaches are impressive, but each has shortcomings. Solvent vapor annealing can lead to polymer chain swelling, longer annealing times, and incompatibility with industrial nanofabrication processes.^19^ The compositions of topcoats must be very carefully tailored to produce a nonpreferential surface for a specific BCP, and the annealing temperature must be lower than the glass transition temperature of topcoats to avoid intermixing. Furthermore, these approaches all require additional complicated processing steps and therefore lead to increased cost. Thus, it would be ideal to achieve a perpendicular orientation solely via industry-friendly thermal annealing.

Methods to obtain Δγ_sub_ = 0 typically involve the use of random copolymers to modify the substrate, which include chain-end functional copolymers,^20^ homopolymer blends,^21^ cross-linkable random copolymers,^22^ self-assembled monolayers,^23,24^ and adsorbed homopolymer chains.^25^ Chain-end-functionalized random copolymers (polymer brushes) consisting of the constituent monomers of the BCP are commonly used because three key parameters, namely molecular weight (*M*_n_), *f*, and grafting density (σ), can be modulated independently.^26^ However, in some cases, it can be difficult or even impossible to synthesize such copolymers due to the distinct reactivities of the constituent monomers. From a technological point of view, a generalized approach regardless of reactivity would be ideal.

Recently, we reported a high throughput approach to realize a library of BCPs that satisfy BCP nanolithography applications.^6^ The key was the use of the A-*block*-(B-*random*-C) (A-*b*-(B-*r*-C)) BRC architecture to impart multiple covarying properties such as χ and γ_air_ into one material. Here, to allow a thorough study of interactions of BCP thin films at the substrate interface, we examined two case studies of model A-*b*-(B-*r*-C) BCPs with Δγ_air_ = 0, which was achieved by controlling the composition ratio of C in the (B*-r-*C) block (φ_1,C_).^27^ Two separate poly(B-*random*-C) random copolymer nanocoatings were used to functionalize the surfaces to mimic the (B*-r-*C) block. The wetting behavior could thus be controlled by varying φ_2,C_.

The two model BCPs with Δγ_air_ = 0 were developed from a single parent polymer, polystyrene*-block-*poly(glycidyl methacrylate) (S*-b-*G), via thiol–epoxy “click” chemistry as reported previously,^6^ to make S-*b*-G(R_B_-*r*-R_C_) BCPs. The nanocoating materials were synthesized by modifying poly(glycidyl methacrylate) (G) homopolymer in a manner similar to the modification of S*-b-*G with the corresponding thiol pairs to make G(R_B_-*r*-R_C_) random copolymers. Two sets of thiol pairs were used: 2-mercaptopyridine (2MP) and trifluoroethanethiol (TFET) as well as methyl thioglycolate (MTG) and TFET. These two pairs were selected because G(TFET) is less polar than S, and both G(2MP) and G(MTG) are more polar than S, making it possible for the final S-*b*-G(R_B_-*r*-R_C_) BCPs to have Δγ_air_ = 0 at the appropriate value of φ_1,C_. One advantage of thiol–epoxy chemistry is that a secondary hydroxy moiety is generated and can be conveniently used to modify the substrate surface. The combination of the S-*b*-G(R_B_-*r*-R_C_) BCPs and their corresponding G(R_B_-*r*-R_C_) random copolymers from the two distinct pairs of thiols effectively created two separate case studies for analyzing BCP–random copolymer interfacial interactions. Case study #1 examined S*-b-*G(TFET-*r*-2MP) on G(TFET-*r*-2MP), and case study #2 examined S*-b-*G(TFET-*r*-MTG) on G(TFET-*r*-MTG). The wetting behaviors of S-*b*-G(R_B_-*r*-R_C_) BCPs with Δγ_air_ = 0 on the corresponding G(R_B_-*r*-R_C_) nanocoatings in the two case studies were investigated to find φ_2,C_ values at which Δγ_sub_ = 0. From a technological perspective, this work demonstrates an efficient and generalized approach to modify the substrate–BCP interaction to yield a perpendicular orientation and could be generalized to other chemistries over a wide range of BCPs with the A*-b-*(B*-r-*C) polymer architecture.

## Experimental Section

### Materials and Syntheses

Styrene (99%, Aldrich), glycidyl methacrylate (GMA, 99%, Aldrich), 2-cyano-2-propyl benzodithioate (CPDB, 97%, Strem Chemicals), 2-mercaptopyridine (2MP, 99%, Aldrich), methyl thioglycolate (MTG, 95%, Aldrich, volatile and with very unpleasant smell!), 2,2,2-trifluoroethanethiol (TFET, 95%, Aldrich, volatile and with very unpleasant smell!), 2,2′-azobis(2-methylpropionitrile) (AIBN, 98%), lithium hydroxide (LiOH, 99.99%, Aldrich), tetrahydrofuran (THF, 99.9%, Fisher Chemical), *N*,*N*-dimethylformamide (DMF, anhydrous, 99.8%, Aldrich), and diiodomethane (CH_2_I_2_, 99%, Aldrich) were purchased and used as received unless otherwise noted. LiOH aqueous solution was freshly made before each thiol–epoxy reaction at a concentration of approximately 20 mg mL^–1^ in deionized water. Inhibitor was removed by passing commercial styrene and GMA monomers through a basic alumina column prior to use. AIBN was recrystallized from methanol twice prior to use.

#### Synthesis of Poly(glycidyl methacrylate) (G) End-Functionalized with the Chain Transfer Agent (CPDB)

To a two-neck round-bottom flask equipped with a condenser and a magnetic stir bar were added GMA (50.0 g, 351.7 mmol), AIBN (0.22 g, 1.3 mmol), and CPDB (0.89 g, 4.0 mmol). After three freeze–pump–thaw cycles, the mixture was stirred at 60 °C for 1 h and then quenched with liquid N_2_. The resulting polymer was purified with three precipitation cycles in hexanes and dried in a vacuum oven overnight.

#### Synthesis of Polystyrene-*block*-Poly(glycidyl methacrylate) (S-*b*-G)

To a two-neck round-bottom flask equipped with a condenser and a magnetic stir bar were added styrene (50.0 g, 351.7 mmol), AIBN (0.22 g, 1.3 mmol), and PGMA–CPDB macro-chain transfer agent (0.89 g, 4.0 mmol). After three freeze–pump–thaw cycles, the mixture was stirred at 60 °C for 1 h and then quenched with liquid N_2_. The resulting polymer was purified by three precipitation cycles in hexanes and dried in a vacuum oven overnight.

#### Standard Procedure for Thiol–Epoxy Modification of S-*b*-G

To a two-neck round-bottom flask equipped with a magnetic stir bar was added a solution of S-*b*-G (116.0 mg, GMA unit 0.33 mmol), 2MP (84 mg, 0.76 mmol, 3.29 equiv to GMA unit), and TFET (22.1 mg, 0.19 mmol, 0.58 equiv to GMA unit) in THF (2.5 g). The solution was then cooled to 0 °C followed by the addition of an aqueous solution of LiOH (40 μL, 0.07 equiv to GMA unit). The reaction was then warmed to room temperature and stirred for 18 h. The product was obtained after three precipitation cycles in hexanes and dried in a vacuum oven overnight. φ_c_, *M*_n_, and dispersity (*Đ*) were characterized with ^1^H nuclear magnetic resonance spectroscopy (^1^H NMR) and size exclusion chromatography (SEC).

### Material Characterization

^1^H NMR was performed on a Bruker AVANCE II+ 500. The NMR samples were dissolved in a deuterated solvent (CDCl_3_) at a concentration of approximately 15 mg mL^–1^. *M*_n_ and *Đ* were characterized with SEC on a Shimadzu gel permeation chromatography system equipped with a Wyatt DAWN HELEOS II multiangle light scattering detector, a Wyatt ViscoStar III differential viscometer, a Wyatt Optilab T-rEX differential refractive index detector, and a Shimadzu SPD-M_30_A photodiode array detector (200–800 nm). The pump used was a Shimadzu HPLC LC20-AD. THF with 250 ppm of BHT was used as the eluent solvent, and the column sets were 2 Agilent PLgel 5 μm MIXED-D plus guard.

Differential scanning calorimetry (DSC) was recorded on a TA Instruments Discovery DSC 2500. The sample was sealed in a hermetic aluminum pan before measurement. The samples were equilibrated at 150 °C for 5 min to eliminate thermal history, then cooled to −80 °C at a rate of 10 °C min^–1^, and equilibrated for another 5 min followed by a heating ramp at a rate of 10 °C min^–1^. The glass transitions were analyzed on the second heating cycle. Thermal gravimetric analysis (TGA) was performed on a TA Instruments Discovery TGA. The sample was placed on a platinum pan and thermally equilibrated at 105 °C for 10 min to remove any possible trapped solvents and moisture followed by a heating ramp to 600 °C at a rate of 10 °C min^–1^ in a N_2_ atmosphere.

Scanning electron microscopy (SEM) images were acquired on a Zeiss Merlin high-resolution field-emission SEM with a 1–1.5 keV accelerating voltage at a working distance below 4 mm using the in-lens secondary electron detector. Image brightness and contrast were adjusted for presentation. Atomic force microscopy (AFM) was performed on a Bruker Nanoscope IIIa Multimode 5 instrument using tapping mode in air. Small-angle X-ray scattering (SAXS) data were collected on a SAXSLAB (Xenocs) Ganesha with a rotating anode (Cu Kα) providing a focused X-ray beam with λ = 0.154 nm. The detector used was a Gabriel-type multiwire area detector (1024 × 1024 pixels).

Grazing-incidence small-angle X-ray scattering (GISAXS) images were collected with a Beamline 8-ID-E at the Advanced Photon Source, Argonne National Lab. Diffraction patterns were collected by using a photon energy of 10.9 keV. An incidence angle of 0.14° was chosen for all reported measurements. This angle was chosen because it is above the typical critical angle of the organic thin film but below the critical angle of the silicon substrate. The exposure time for the measurements was 3–10 s. The images were collected at a sample-to-detector distance of 2185 mm using a Pilatus MF pixel array detector. To minimize beam damage and background air scatter, measurements were performed in a vacuum chamber at ∼10^–3^ Torr.

### Nanocoating Preparation

Nanocoatings were prepared by spin-coating the nanocoating solution onto silicon wafers purchased from Pure Wafer, which were precleaned using a hot piranha solution (a mixture of 30% H_2_O_2_ and 70% (v/v) concentrated H_2_SO_4_; *use with caution*!) and rinsed with deionized H_2_O. The nanocoatings were then annealed in a glovebox at 150 °C for 1 h. After annealing, the wafer was sonicated by using alternating rinses with THF and a THF/DMF mixture three times to remove any unattached polymer.

### Metrology

Film thicknesses were measured using a J.A. Woollam Alpha SE ellipsometer and fitted using a Si-SiO_*x*_-Cauchy model, where the native oxide thickness was preset at 1.5 nm. For films with thickness <10 nm, the optical constants were first fit to thick films and then locked to capture the film thickness more accurately.

### Surface Energy (γ) Calculation

Surface energy (γ) values were determined from contact angle measurements, which were performed with a KRÜSS drop shape analyzer DSA100 (KRÜSS GmbH). Solutions of the nanocoating in PGMEA or THF were spin-coated on a piranha-cleaned silicon wafer to form thin films with a thickness of ∼30 nm. The thin films were then annealed on a hot plate at 150 °C for 1 h inside a N_2_ glovebox. Before each contact angle measurement, dry N_2_ was blown over the film surface to remove the particle contaminants. The contact angles of two probing liquids, deionized H_2_O and CH_2_I_2_, were recorded by using the sessile drop method with a drop volume of 1 μL for each measurement. The left and right contact angles of each drop were averaged, and 10 sessile drops were deposited for each sample. γ was then calculated using the OWRK method.^28,29^

## Results and Discussion

**Figure 1:**
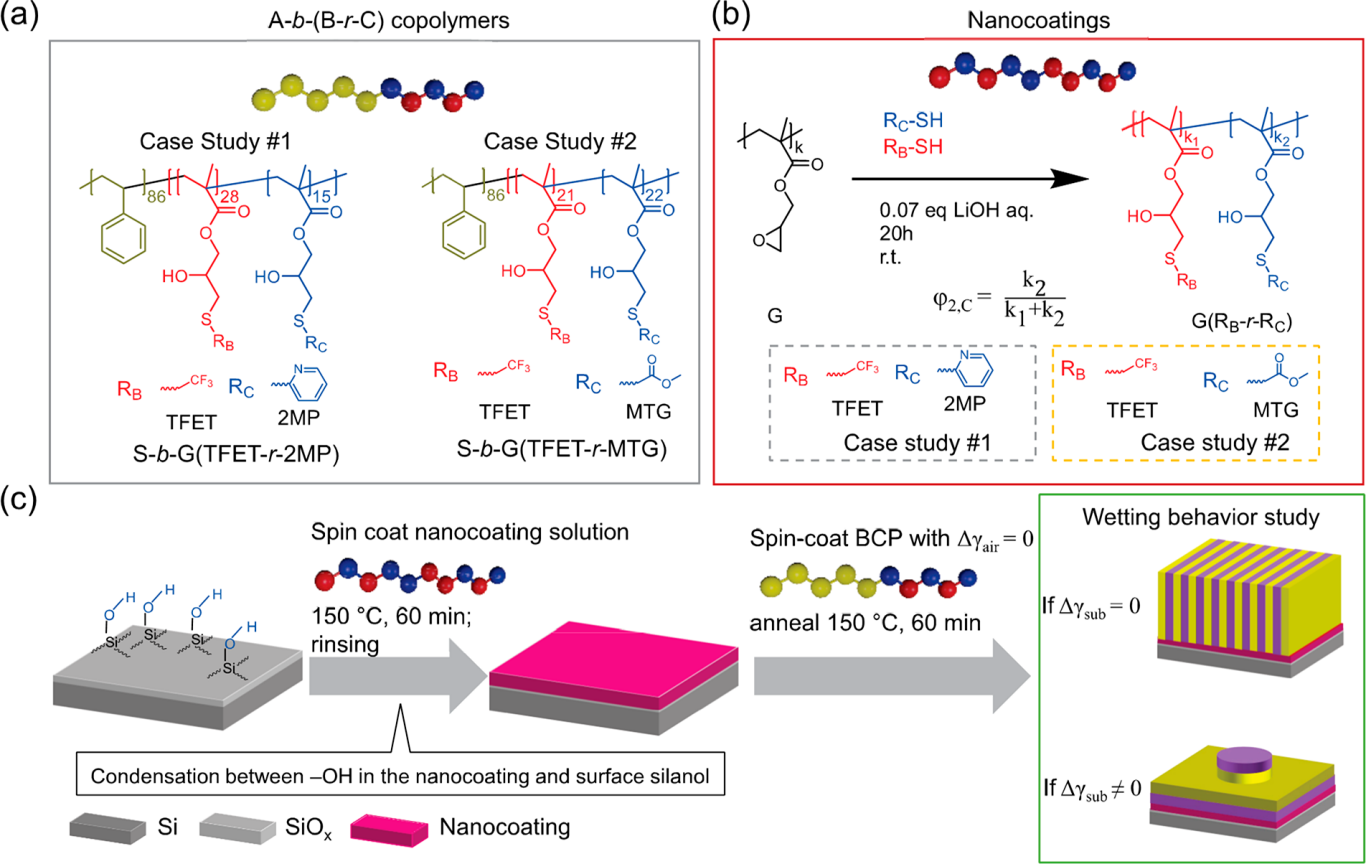
(a) Schematics of the model A-*b*-(B-*r*-C) BCPs with Δγ_air_ = 0 for case studies #1 and #2 with the corresponding average number of repeat units for each BCP. (b) Synthesis of the corresponding B-*r*-C nanocoatings for the two case studies with different ratios of R_C_ (φ_2,C_). (c) Schematic of the process flow to determine if the difference in interfacial energy between each of the blocks in the BCP and the nanocoating (Δγ_sub_) equals 0. Self-assembly of the BCP on nanocoatings with φ_2,C_ that cause Δγ_sub_ = 0 results in the formation of a perpendicular lamellae.

Figure 1 outlines the approach used in the two case studies, including the chemical structures of the two model S-*b*-G(R_B_-*r*-R_C_) BCPs and the corresponding G(R_B_-*r*-R_C_) random copolymers, the experimental flow of coating the substrates with the G(R_B_-*r*-R_C_) to make nanocoatings, subsequent wetting behavior studies, and the formation of perpendicular or parallel self-assembled BCP domain orientations, depending on Δγ_sub_. In the thiol pairs used in case studies #1 and #2, 2MP and MTG served as the polar thiol, respectively, and TFET served as the nonpolar thiol in both case studies. After the thiol–epoxy reaction, a secondary hydroxyl group (−OH) was automatically generated for each epoxy unit in the nanocoating. The substrate could then be modified via a condensation reaction between this −OH group and the surface silanol group (−Si–OH). The value γ_sub_ was changed by varying the composition ratio of 2MP (φ_2,2MP_) or MTG (φ_2,MTG_).

First, G was synthesized via reversible addition–fragmentation chain transfer (RAFT) polymerization techniques using the CPDB as the chain transfer agent. The obtained G had a reasonably narrow *Đ*. The synthesis of the model BCPs in each case study, S*-b-*G(TFET*-r-*2MP) and S*-b-*G(TFET*-r-*MTG) with Δγ_air_ = 0, can be found in the literature.^6^ Achieving Δγ_air_ = 0 in the two models BCPs required that φ_1,2MP_ = 0.357 for S*-b-*G(TFET*-r-*2MP) and φ_1,MTG_ = 0.515 for S*-b-*G(TFET*-r-*MTG). The nanocoating materials for case studies #1 and #2, G(TFET*-r-*2MP) and G(TFET*-r-*MTG), respectively, were synthesized by modifying homopolymer G with the same thiol pairs in the same way the corresponding BCP was thiol-functionalized (Figure 1b). Values of φ_2,C_, *M*_n_, and *Đ*, which were determined with ^1^H NMR spectroscopy and SEC, can be found in the Supporting Information (Figures S1 and S2 and Table S1). To determine proper processing temperatures of the coating materials, the thermal properties were characterized as shown in Figure S3a,b.

**Figure 2:**
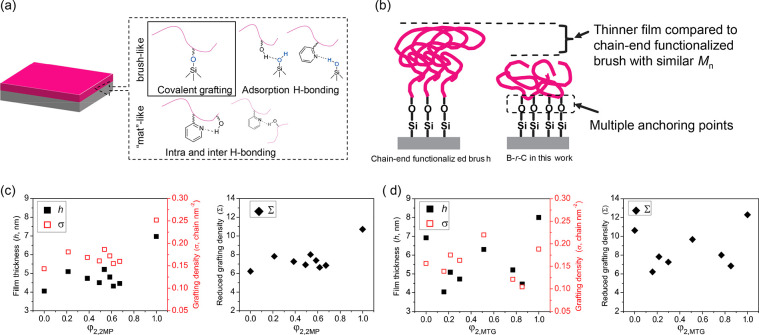
Characterization of substrates coated with G(TFET-*r*-2MP) or G(TFET-*r*-MTG). Both random copolymers were grafted to the silicon substrate by heating at 150 °C for 1 h followed by three sonication cycles in DMF for 5 min per cycle to remove ungrafted material. (a) Schematics showing possible interactions between the silicon substrate and the copolymers. Both brushlike and matlike behaviors could occur due to covalent grafting and hydrogen-bonding (H-bonding) interactions. After the mixture is rinsed with DMF, which disrupts the H-bonding interaction, covalent grafting should be the dominant interaction. (b) Schematic comparison of the bonding of a G(R_B_-*r*-R_C_) random copolymer, with multiple grafting sites, to a standard end-functionalized random copolymer brush. (c) Left: film thickness (left *y*-axis, solid black square) and grafting density (right axis, open red square) of G(TFET-*r*-2MP) as a function of φ_2,2MP_. Right: reduced grafting density (Σ) as a function of φ_2,2MP_. (d) Left: film thickness (left *y*-axis, solid black square) and grafting density (right axis, open red square) of G(TFET-*r*-MTG) as a function of φ_2,MTG_. Right: reduced grafting density as a function of φ_2,MTG_.

To evaluate the chain conformation of these nanocoatings, a series of G(TFET*-r-*2MP) and G(TFET*-r-*MTG) thin films with φ_2,2MP_ and φ_2,MTG_ values ranging from 0 to 1 are prepared. Each repeat unit of these random copolymers contains one secondary hydroxyl group that can undergo a condensation reaction with a surface silanol group^30−32^ or form hydrogen bonds with either surface silanols, other repeat units in its polymer chain (intra-H-bonding), or other polymer chains (inter-H-bonding), as shown in Figure 2a. Most noncovalent bonds, such as hydrogen bonds, can be readily disrupted, such that rinsing with the appropriate solvent will leave behind only the covalently bonded materials (i.e., Si–O–R groups). However, it has been shown that the reactivity of a secondary hydroxyl is lower than a primary hydroxyl.^33,34^ Given the lower reactivity as well as competition with H-bonding, it is reasonable to assume that only part of the −OH groups of the G(R_B_-*r*-R_C_) nanocoating react with the surface Si–OH, while another portion of the −OH of the G(R_B_-*r*-R_C_) forms a hydrogen bond (H-bond) network. The conformation of the nanocoating is assumed to be close to the case of a polymer brush but with multiple anchoring points between the polymer chain and the substrate, resulting in a thinner polymer film, as shown in Figure 2b.

The conformation of the polymer chain can be assessed using the grafting density (σ) and reduced grafting density (Σ).^31,35^ Both parameters can be calculated with^36^
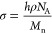
1
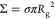
2where *h* is the thickness of the thin film, ρ is the density of the polymer (ρ = 1.00 g cm^–3^ is used in this work), and *N*_A_ is Avogadro’s number. *R*_g_ is the radius of gyration of the polymer chain, which can further be estimated with
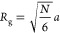
3where *a* is the statistical segment chain length, which is estimated to be 0.65 nm in this work.^37^

Figure 2c shows *h* and σ as a function of φ_2,2MP_ of the G(TFET*-r-*2MP) nanocoating. These nanocoatings have very similar *M*_n_ values of ∼17.0 kg mol^–1^ and a thickness of 5.1 nm compared to the as-cast thickness of 10 nm. The σ has a value of ∼0.15–0.2 chains/nm^2^, which is slightly lower than half of the reported σ for two different molecular weights of a chain-end hydroxyl-terminated poly(styrene*-random-*methyl methacrylate) random copolymer (P(S*-r-*MMA)–OH) brush annealed at very similar conditions, with σ = 0.41 for the 14.1 kg mol^–1^ polymer and 0.37 for the 19.9 kg mol^–1^ polymer.^26^ As a comparison, the surface silanol has a density of 4.6 ± 0.2 Si–OH/nm^2^ after a similar piranha solution treatment.^38^ This suggests that the polymer brush may have a bending conformation (Figure 2b). The polymer is also capable of forming multiple anchoring points to the substrate. A similar trend has been previously observed in the side-chain-grafted copolymer poly(styrene*-random-*methyl methacrylate*-random-*2-hydroxyethyl methacrylate) P(S*-r-*MMA*-r-*HEMA), in which graftable hydroxyl groups were assumed to be randomly distributed along the polymer side chain.^32^ After thermal annealing, the polymer brush with the most HEMA content (more grafting sites) has the thinnest film, indicating a large number of covalent anchors to the silicon surface. Additionally, as shown in Figure 2b,d, the calculated Σ of the nanocoatings is >5, indicating that the polymer chain is highly stretched into a brush regime.^39^ It is thus reasonable that the interaction between BCP nanodomains and the Si surface is sufficiently shielded. Therefore, the wetting behavior between the nanocoating and the BCP domains depends on φ_2,C_.

**Figure 3:**
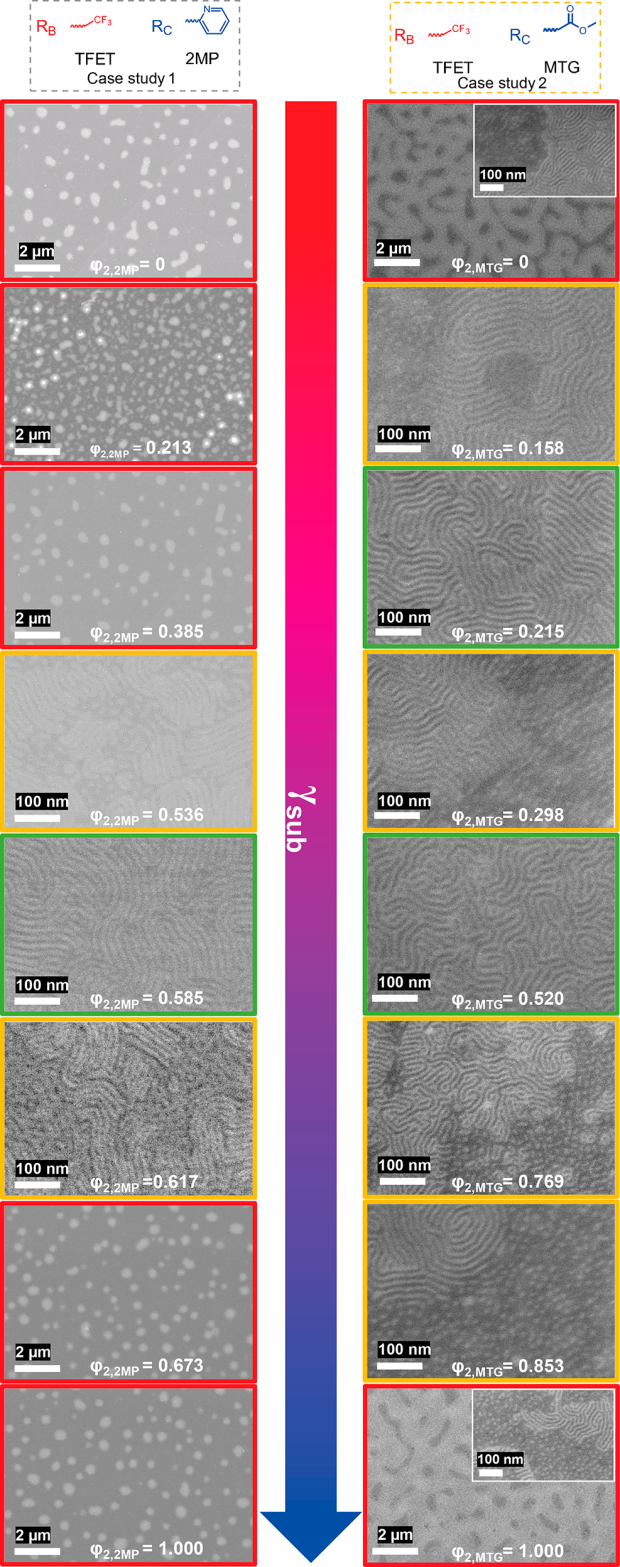
Representative top-down SEM images showing different wetting behaviors of the two case studies. Left column: case study #1, thin films of S*-b-*G(TFET-*r*-2MP) with Δγ_air_ = 0 on G(TFET-*r*-2MP) with different values of φ_2,2MP_. Right column: case study #2, thin films of S*-b-*G(TFET-*r*-MTG) with Δγ_air_ = 0 on G(TFET-*r*-MTG) with different values of φ_2,MTG_. The thickness of BCP thin films was ∼1.7*L*_0_ (27 nm) and was annealed at 150 °C for 1 h.

As shown in the top-down SEM images of the two material case studies in Figure 3, in case study #1, S*-b-*G(TFET-*r*-2MP) on G(TFET-*r*-2MP), typical island–hole features were present on the substrates with φ_2,2MP_ ≤ 0.385 or φ_2,2MP_ ≥ 0.673, while fingerprints were observed on nanocoatings with 0.536 ≤ φ_2,2MP_ ≤ 0.617. When φ_2,2MP_ = 0.536 and 0.617, features including short lines and dots were observed, indicating the presence of a mixed orientation.^39^ The large fingerprint area observed when φ_2,2MP_ = 0.585 indicated that the lamellae had assembled perpendicular to the substrate, and therefore Δγ_sub_ = 0 was achieved. It is worth noting that there is a difference in the values of φ_1,2MP_ in the BCP (φ_1,2MP_ = 0.357) for Δγ_air_ = 0 and φ_2,2MP_ of the nanocoating (φ_2,2MP_ = 0.536–0.617) for Δγ_sub_ = 0. This difference in φ_C_ values simply reflects that φ_1,2MP_ and φ_2,2MP_ are tailored to achieve two different effects. φ_1,2MP_ is used to make γ_air_ of S and G(TFET-*r*-2MP) in the BCP equal, whereas φ_2,2MP_ is adjusted so that γ_sub_ between G(TFET-*r*-2MP) and the two blocks of the BCP are equal. In case study #2, with S*-b-*G(TFET-*r*-MTG) on G(TFET-*r*-MTG), a mixed orientation rather than a distinct island–hole feature was observed with φ_2,MTG_ = 0, which persisted until φ_2,MTG_ = 0.215, where a large area of fingerprint appeared. Very interestingly, a mixed orientation appeared again as φ_2,MTG_ further increased until reaching φ_2,MTG_ = 0.520, where a large area of the fingerprint appeared again. At even higher φ_2,MTG_ the mixed orientation re-emerged. Thus, in case study #2, there were two windows of φ_2,MTG_ values that resulted in Δγ_sub_ = 0.

**Figure 4:**
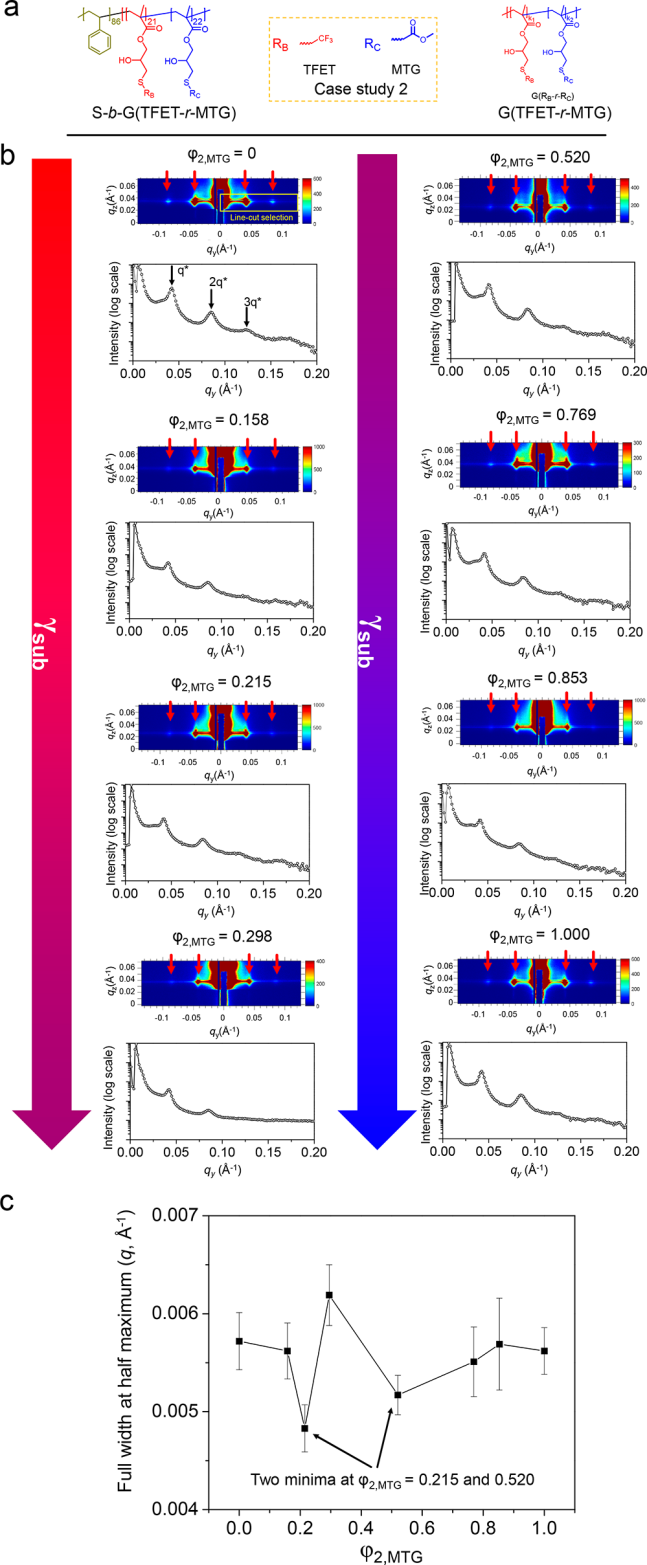
(a) Chemical structures of BCP, S*-b-*G(TFET-*r*-MTG), and nanocoating, G(TFET-*r*-MTG), in case study #2. (b) GISAXS profile including 2D pattern and corresponding line-cut one-dimensional profile of S*-b-*G(TFET-*r*-MTG) thin film on G(TFET-*r*-MTG) with different values of φ_2,MTG_. The downward pointing arrows indicate that γ_sub_ increases continually as φ_2,MTG_ increases from 0 to 1. (c) Plot of full width at half-maximum of principal peaks of GISAXS profiles as a function of φ_2,MTG_. Two minima are presented that agree well with the SEM analysis in Figure 3.

GISAXS was used to verify that there were two windows of φ_2,MTG_ values that resulted in Δγ_sub_ = 0 in case study #2 and to enable a macroscopic, quantitative investigation, as shown in Figure 4b. The 2D profile indicated the presence of in-plane ordering (perpendicular orientation). The parallel orientation (out-of-plane) was difficult to observe, most likely because the orientation was mainly in-plane. The full width at half-maximum of the one-dimensional profile of a selected region (in-plane orientation) (Figure 4c) had two minima, at φ_2,MTG_ = 0.215 and 0.520, which agreed with the two φ_2,MTG_ windows that generated fingerprint patterns in the SEM images.

**Figure 5:**
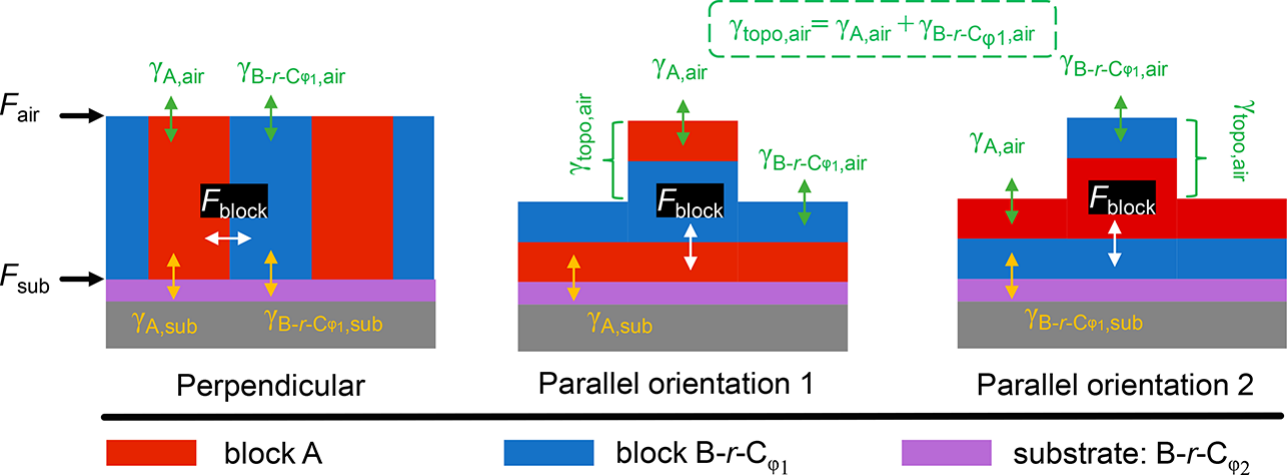
Illustration of interfacial energy components of BCP self-assembly with perpendicular and parallel orientations for BCPs whose blocks have equal surface energies, i.e., γ_A,air_ = γ_B-*r*-Cφ1,air_. The as-cast film thickness is 0.75*L*_0_ to simplify the analysis, but the methodology can be extended to other thicknesses. For the perpendicular domain assembly, the blocks also have interfacial energies equal to those of the substrate (γ_A,sub_ = γ_B-*r*-Cφ1,sub_). For parallel orientation 1, γ_A,sub_ < γ_B-*r*-Cφ1,sub_, and for parallel orientation 2, γ_A,sub_ > γ_B-*r*-Cφ1,sub_.

The existence of two windows of φ_2,MTG_ values that result in Δγ_sub_ = 0 can be explained by analyzing the thermodynamics of the system. If a thin film of symmetric BCP, in which the volume fraction of the two blocks of the BCP are equal, is in a thermodynamically equilibrated state, the orientation of the lamellae, either parallel or perpendicular, is governed by the minimization of total free energy of the system, which is the sum of the entropic free energy associated with the limited extensibility of each chain (*F*_ent_) and the interfacial energies (per unit chain) at the free surface (*F*_air_), the substrate interface (*F*_sub_), and the block–block interface (*F*_block_).^40^ In a comparison of different self-assembly orientations of the same volume of BCP at the same temperature, *F*_ent_ is constant and can be ignored. *F*_air_, *F*_sub_, and *F*_block_ depend on the interfacial energy of each block with each surface (denoted as γ_*x*,*y*_, where *x* is the block and *y* is the surface, e.g., γ_B-*r*-C,sub_ for the B*-r*-C block in contact with the substrate) and the area of each block at the surface (denoted as *A*_*x*,*y*_, with the same meanings for *x* and *y* as for γ_*x*,*y*_). These interfacial energies for different orientations are illustrated in Figure 5.

For the perpendicular orientation
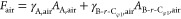
4
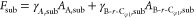
5
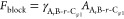
6

For parallel orientation 1
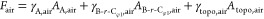
7
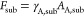
8
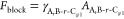
9

For parallel orientation 2
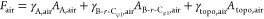
10
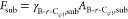
11
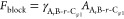
12where γ_A,B-*r*-Cφ1_ and *A*_A,B-*r*-Cφ1_ are the interfacial energy and area, respectively, at the interface of A and B-*r*-C_φ1_, and γ_topo,air_ and *A*_topo,air_ are the interfacial energy and area, respectively of the sides of the topographic feature. In an ideal case, γ_topo,air_ is effectively the sum of γ_A,air_ and γ_B-*r*-C_φ1_,air_.

It is worth noting that at equilibrium for a fixed volume of BCP, *F*_block_ in all three domain orientations is constant and independent of the domain orientation. For a symmetric BCP having Δγ_air_ = 0 (i.e., γ_A,air_ = γ_B-*r*-C_φ1_,air_) with a perpendicular orientation and blocks with equal density, *A*_A,air_, *A*_B-*r*-C_φ1_,air_, *A*_A,sub_, and *A*_B-*r*-Cφ1,sub_ are identical and are each half of the area of the substrate. For both parallel orientations, for a film with thickness (*n* + 0.75)*L*_0_ (*n* is an integer), *A*_A,air_ = *A*_B-*r*-C_φ1_,air_ = 0.5*A*_A,sub_ (parallel orientation 1) and *A*_A,air_ = *A*_B-*r*-C_φ1_,air_ = 0.5*A*_B,sub_ (parallel orientation 2). Therefore, *F*_air_ is constant, which in fact holds true for BCP thin films with any as-cast thickness. Thus, the difference in total free energy is dominated by *F*_sub_ and the additional free energy involved in the creation of the new surfaces of the topographical features, *F*_topo_ = γ_topo,air_*A*_topo,air_. In both cases of parallel orientation, *F*_topo_ is identical.

If the substrate interaction is nonpreferential (γ_A,sub_ = γ_B-*r*-C_φ1_,sub_), then *F*_A,sub_ = *F*_B-*r*-C_φ1_,sub_, and the minimization of the free energy of the system requires that *F*_topo_ = 0, which can only happen when the domains have a perpendicular orientation (a flat surface without any topographical features). Thus, in the case of a nonpreferential treatment, the perpendicular orientation always has the lowest free energy and is preferred at equilibrium, which has previously been explained by restrictions on polymer chain configurations that cause BCP chains to arrange themselves parallel to nonpreferential surfaces.^41,42^ When γ_A,sub_ and γ_B-*r*-C_φ1_,sub_ are similar but not equal, a perpendicular domain orientation can still be achieved for a certain range of film thicknesses because of the balance of *F*_topo_ (determined by the film thickness) and *F*_sub_. For BCP (with Δγ_air_ = 0) film thicknesses with (*n* + 0.25)*L*_0_, the energy expression in parallel assembly is equivalent to that of films with thicknesses of (*n* + 0.75)*L*_0_. For BCP thicknesses of *nL*_0_ and (*n* + 0.5)*L*_0_, complicated topographic features such as a bicontinuous topography or mixed parallel and perpendicular orientation could occur.^40^ The simplified treatment in this work only considers either parallel or perpendicular orientation, but the conclusion holds that for any given BCP film thickness, perpendicular assembly, which eliminates *F*_topo_ and requires the blocks to have equal interfacial energies with the substrate, has the smallest free energy.

In the case of the desired perpendicular orientation, the substrate interface interaction can be expressed using the following equations:
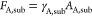
13
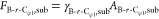
14Assuming a symmetric BCP, *A*_A,sub_ and *A*_B-*r*-C_φ1_,sub_ are equal and therefore γ_A,sub_ = γ_B-*r*-C_φ1_,sub_. Because the substrate used in this work has the same chemical components to the block, the only difference being between the B*-r-*C_φ1_ and the B-*r*-C_φ2_ substrate coating is the ratio of C in each material, φ_1_ and φ_2_. Thus, the interfacial interactions between the A block and the B-*r*-C_φ2_ (substrate) and between B-*r*-C_φ1_ (the other block in the BCP) and B-*r*-C_φ2_ (substrate) can be treated like block–block interfacial interactions. Following classic lattice–lattice interaction relationships gives^43^
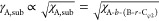
15
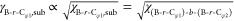
16where χ_A,sub_ is equivalent to the χ of a BCP consisting of A and B-*r*-C_φ2_, (i.e., A*-b-*(B-*r*-C_φ2_)), and χ_B-*r*-Cφ1,sub_ is equivalent to the χ of a BCP consisting of B-*r*-C_φ1_ and B-*r*-C_φ2_, (i.e., (B-*r*-C_φ1_)*-b-*(B-*r*-C_φ2_)). The use of a binary interaction model gives the following relationships:^44^
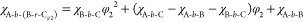
17
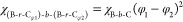
18The interfacial tension expressions (15) and (16) can thus be rearranged as
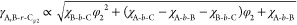
19
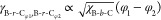
20

**Figure 6:**
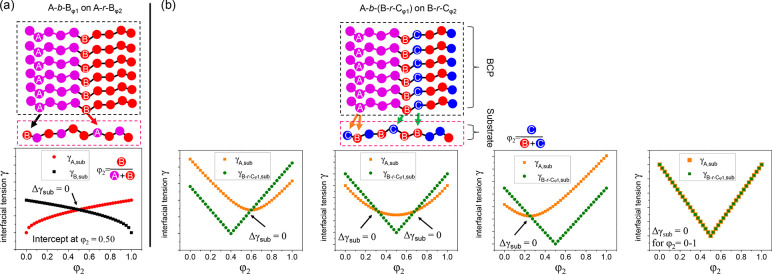
Schematic of interfacial tension between the perpendicular domains of a symmetric BCP and a polymer-coated substrate for two types of BCP architectures. (a) For A*-b-*B on A*-r-*B (e.g., PS*-b-*PMMA on a P(S-*r*-MMA) brush), the predicated nonpreferential φ_2_ of the substrate is 0.50. (b) For A*-b-*(B*-r-*C) on B*-r-*C, due to the possible different landscape of the χ–φ_2_ relationship there are different possibilities for the relationship between interfacial tension γ and φ_2_. Here two possibilities are shown: γ_A,sub_ (orange dots) and γ_B*-r-*C,sub_ (green dots) are plotted as a function of φ_2_. In one particular scenario, γ_A,sub_ and γ_B*-r-*C,sub_ can overlap completely, indicating that Δγ_sub_ = 0 for φ_2_ = 0–1. This is illustrated in the bottom right figure, where the solid orange squares representing γ_A,sub_ are enlarged for visualization.

The interfacial tensions at the substrate interface for both a standard A*-b-*B BCP on an A*-r-*B random copolymer brush, like polystyrene-*block*-poly(methyl methacrylate) (PS-*b*-PMMA) on P(S-*r*-MMA), and for the A-*b*-(B-*r*-C_φ1_) BCP on the B-*r*-C_φ2_ brush studied in this work are illustrated in Figure 6. It should be noted that the value of φ_1_ in the BCP is determined by different thiol pairs to reach Δγ_air_ = 0, where the methodologies have been experimentally verified.^6,27,45,46^ For the A-*b*-B BCP in Figure 6a, there is one value of φ_2_ where γ_A,sub_ = γ_B,sub_. For the A-*b*-(B-*r*-C_φ1_) BCP on the B-*r*-C_φ2_ brush the curve of γ_A,B-*r*-Cφ2_ vs φ_2_ is parabola-shaped (eq 19), with a minimum when φ_2_ = 0.55 (Figure 6b). In contrast, the curve of γ_B-*r*-C_φ1_,B-*r*-C_φ2__ vs φ_2_ is “V-shaped” (eq 20) and approaches zero when φ_1_ = φ_2_ = 0.357. This is because when φ_1_ = φ_2_, the B*-r-*C block and the substrate are chemically identical, and zero interfacial tension should be expected. Because of the difference between the parabolic shape of the γ_A,B-*r*-Cφ2_ curve and the V shape of the γ_B-*r*-C_φ1_,B-*r*-C_φ2__ curve, it is possible to have two values of φ_2_ at which γ_B-*r*-C_φ1_,B-*r*-C_φ2__ = γ_A,B-*r*-C_φ2__, Δγ_sub_ = 0, and a nonpreferential surface is achieved. Following this analysis, due to different χ–φ landscapes provided by the different A, B, and C chemistries of the A-*b*-(B-*r*-C) architecture, other γ–φ landscapes are also possible, as illustrated in Figure 6b.

**Figure 7:**
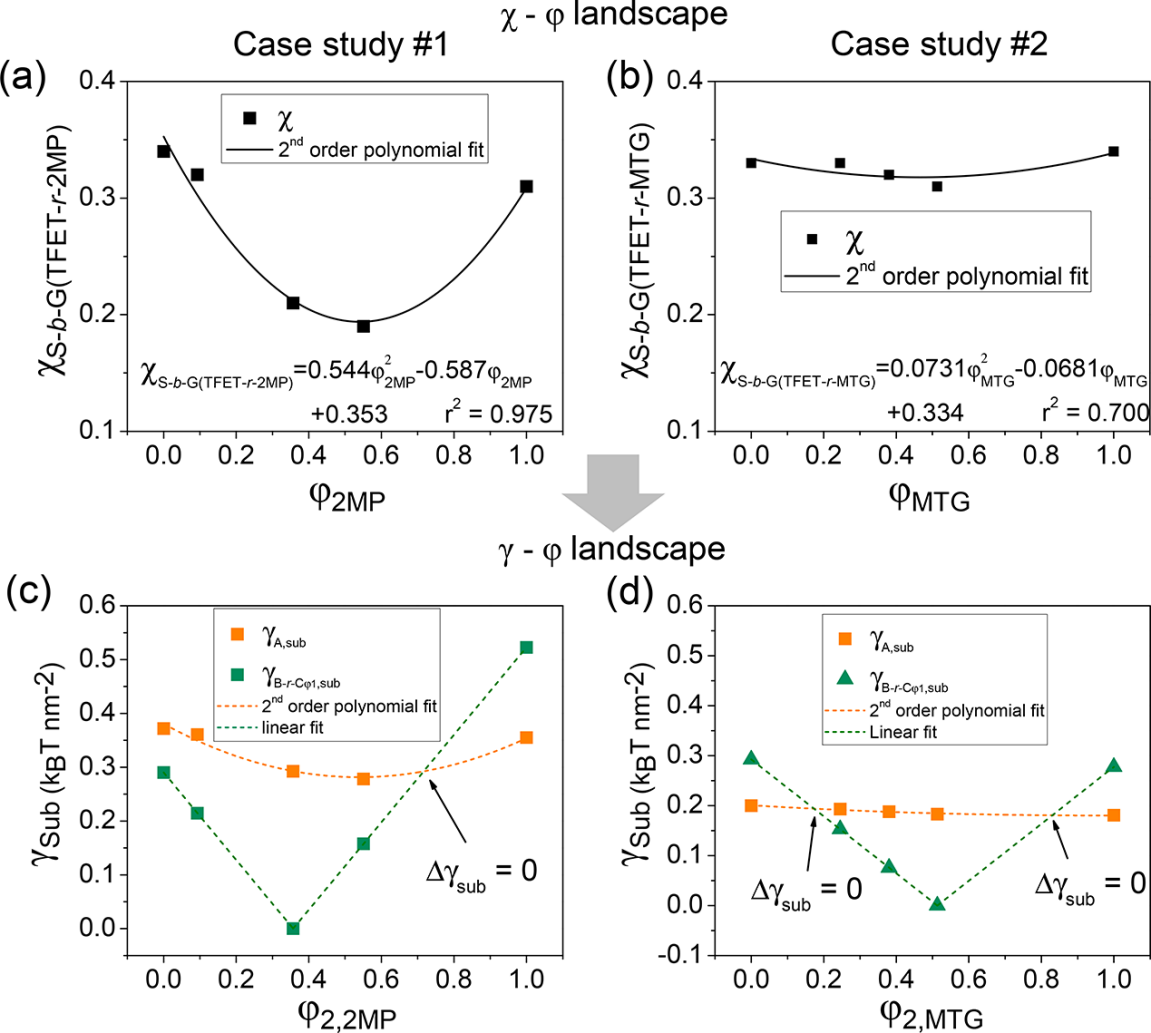
χ–φ and γ–φ landscapes for the two case studies to illustrate the distinct wetting behaviors. (a) Plot of χ_S*-*b*-*G(TFET-*r*-2MP)_ as a function of φ_2MP_ for case study #1. (b) Plot of χ_S*-*b*-*G(TFET-*r*-MTG)_ as a function of φ_MTG_ for case study #2. (c) The substrate interfacial energies of γ_A,sub_ (orange dots, orange dashed lines) and γ_B*-r-*Cφ_1_,sub_ (green triangles and green dashed lines) for case study #1. (d) The substrate interfacial energies of γ_A,sub_ (orange dots, orange dashed lines) and γ_B*-r-*Cφ_1_,sub_ (green triangles and green dashed lines) for case study #2. The intersections of the dashed orange and green lines represent Δγ_sub_ = 0. In (d), the value of γ_A,sub_ varies little (∼0.2 *k*_B_*T* nm^–2^), leading to the existence of two values of φ_2,C_ at which Δ_sub_ = 0.

Figure 7 shows the application of the analysis to both case studies using the real χ–φ relationships of S*-b-*G(TFET-*r*-2MP) and S*-b-*G(TFET-*r*-MTG) with χ and φ values determined in previous work.^6^ Plots of χ_S*-*b*-*G(TFET-*r*-2MP)_ as a function of φ_2,2MP_ and χ_S*-*b*-*G(TFET-*r*-MTG)_ as a function of φ_2,MTG_ describe the two χ–φ landscapes, as shown in Figure 7a,b. The γ–φ landscapes are shown in Figure 7c,d. For case study #1, it can be seen that γ_B*-r-*C,sub_ = 0 when φ_2,2MP_ = φ_1,2MP_ with Δγ_air_ = 0 (shown in green dashed line). γ_A,sub_, on the other hand, follows a pseudo-parabolic shape with respect to φ_2,2MP_ (orange dashed line), which has a minima at φ_2,2MP_ of ∼0.55. When φ_2,2MP_ approaches ∼0.60, the two dashed lines intersect, indicating Δγ_sub_ = 0, where a nonpreferential wetting behavior is expected as seen in the SEM results (Figure 3). It is also predicted from Figure 7 that when φ_2,2MP_ < ∼0.60, γ_B*-r-*C,sub_ < γ_A,sub_, implying the modified PGMA block should wet the substrate, and when φ_2,2MP_ > ∼0.60, γ_B*-r-*C,sub_ > γ_A,sub_, implying the PS block should wet the substrate. For case study #2, with S*-b-*G(TFET-*r*-MTG), there were two possible intersections of the χ–φ and γ–φ curves, which was verified by the findings in the SEM images and GISAXS profiles.

The treatment here, of course, is idealized and simplified. Any variations, such as the film thickness, annealing temperatures, and inhomogeneity of the substrate (e.g., composition aggregation, roughness), will have an impact on the landscape of the free energy terms and thus affect the nonpreferential window.^40,47^ Nevertheless, this analysis agrees well with the SEM image analysis and the GISAXS results for case #2. It is likely that continued work with this approach and the unique capability of controlling the χ–φ landscape, and thus the γ_sub_–φ landscape, with the A-*b*-(B-*r*-C) architecture will generate more new, interesting, and potentially helpful wetting behaviors.

## Conclusions

In this work, we demonstrated that polymer chains composed of the (B-*r*-C) block of an A-*b*-(B-*r*-C) BCP can be used as a generalized and efficient surface modification approach to induce industry-desired perpendicularly oriented nanodomains in thin films of the BCP. Two thiol pairs, TFET/2MP and TFET/MTG, were used to modify the parent BCP. The grafting density of the B-*r*-C polymer brush was estimated to be in the range of 0.2 chain/nm^2^, and the dry film thickness had a linear dependence on the grafting density, indicating that the (B-*r*-C) copolymer was in the “brush” regime. Different mole fractions of component C in the BCP and brush coating were necessary to achieve Δγ_air_ = 0 (at φ_1,2MP_ = 0.357) and γ_sub_ = 0 (at φ_2,2MP_ = 0.585) in the nanocoating for γ_sub_ = 0. In the case of TFET/MTG, two φ_2,MTG_ composition windows existed for γ_sub_ = 0, which could be explained by BCP thermodynamics. From a technological point of view, we believe this work will provide a generalized substrate modification method for achieving perpendicular domains in self-assembled BCP films with the A-*b*-(B-*r*-C) architecture. The thermodynamics approach may shed light on understanding of polymer–polymer interactions and orientation controls in BCP thin film assembly.

## Supporting Information Available

^1^H NMR spectra of G(TFET-*r*-2MP) and G(TFET-*r*-MTG) in CDCl_3_ (Figure S1), SEC profiles of G(TFET-*r*-2MP), G(TFET-*r*-MTG), and G in THF (Figure S2), differential scanning calorimetry profiles and thermal gravimetric analysis of the nanocoatings (Figure S3), and summarized characteristics of the two sets of substrates used in the two case studies (Table S1) (PDF)
