# Supplementary material for: Wetting Behavior of A-block-(B-random-C) Copolymers with Equal Block Surface Energies on Surfaces Functionalized with B-random-C Copolymers
Source: Langmuir. 2023 Oct 2;39(41):14688–98. doi: 10.1021/acs.langmuir.3c02065 (PMC10586369; doi:10.1021/acs.langmuir.3c02065)
Supplement: Supplementary file 1 — la3c02065_si_001.pdf [file la3c02065_si_001.pdf]

Supporting Information

Wetting Behavior of A-*block*-(B-*random*-C) Copolymers with Equal Block Surface Energies on Surfaces Functionalized with B-*random*-C Copolymers

Hongbo Feng[1†], Benjamin Kash[1†], Soonmin Yim[1], Kushal Bagchi[1], Gordon S. W. Craig[1], Wen Chen[1], Stuart J. Rowan[1,2,3], and Paul F. Nealey[1,4]*

[1] Pritzker School of Molecular Engineering, University of Chicago, 5640 S. Ellis Avenue, Chicago, Illinois 60637, United States

[2] Department of Chemistry, University of Chicago, 5735 S. Ellis Avenue, Chicago, Illinois 60637, United States

[3] Chemical Sciences and Engineering Division, Argonne National Laboratory, 9700 S. Cass Avenue, Lemont, Illinois 60439, United States

[4] Center for Molecular Engineering, Materials Science Division, Argonne National Laboratory, 9700 S. Cass Avenue, Lemont, Illinois, 60439, United States

*Email: hfeng9@vols.utk.edu

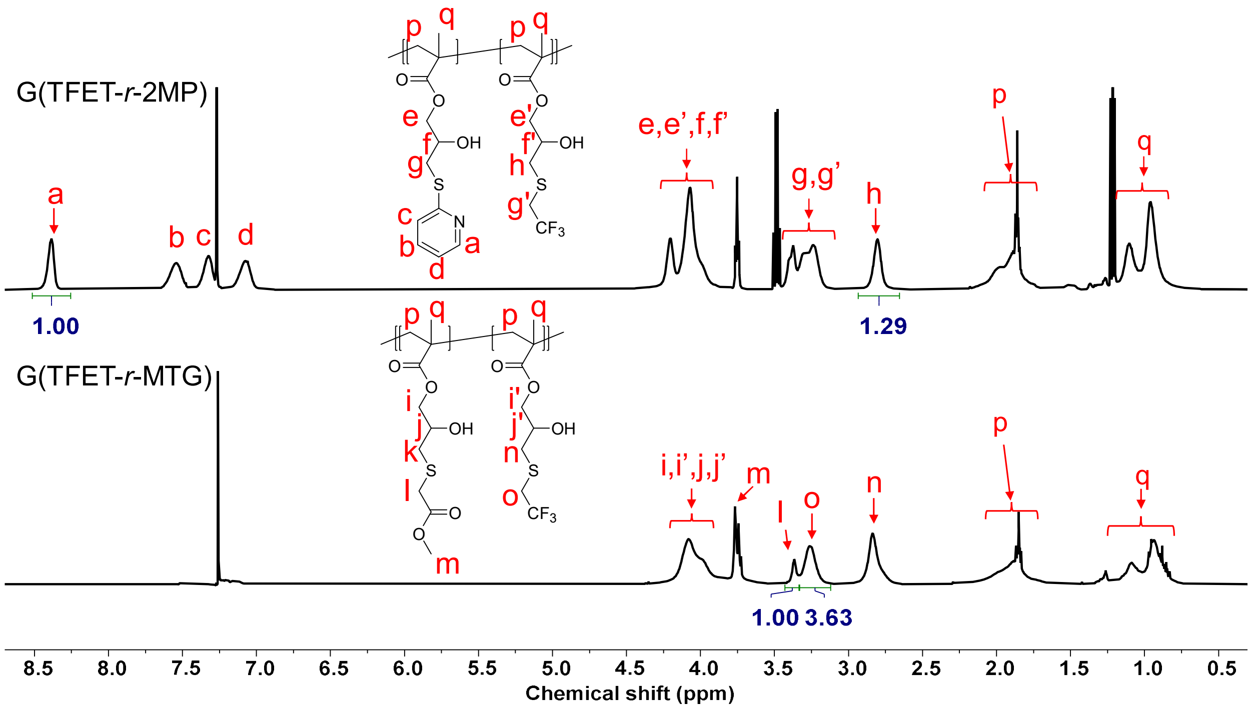

Figure S1. ^{1}H-NMR spectra of G(TFET-*r*-2MP) and G(TFET-*r*-MTG) in $CDCl_3$. The characteristic peaks of the functionalities are labelled.

The calculation of φ_{2MP} and φ_{MTG}.

$$\varphi_{2MP} = \frac{\delta_{2MP}}{\delta_{2MP}+2\times\delta_{TFET}} = \frac{1.00}{1.00+2\times1.29} = 0.279$$

$$\varphi_{MTG} = \frac{2\times\delta_{MTG}}{2\times\delta_{MTG}+2\times\delta_{TFET}} = \frac{2\times1.00}{2\times1.00+2\times3.63} = 0.216$$

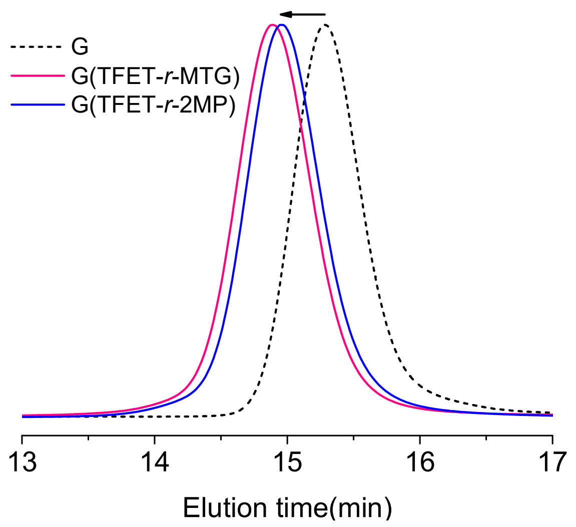

Figure S2. SEC profiles of G(TFET-*r*-2MP), G(TFET-*r*-MTG), and G in THF. The clean shift indicates the thiol-epoxy reaction occurred without side reactions.

Thermal properties of nanocoatings.

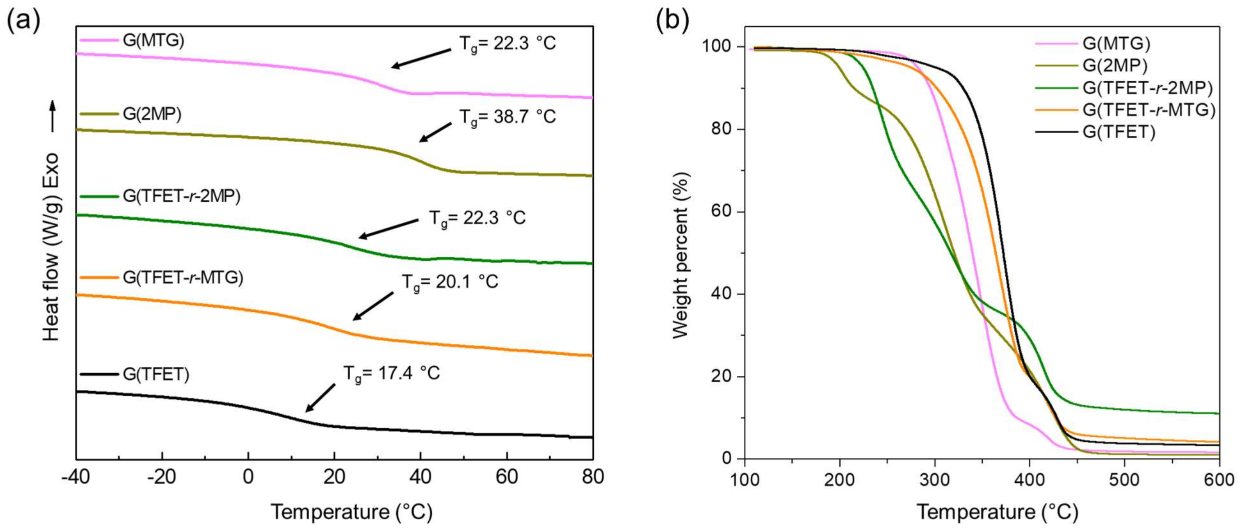

Figure S3. a) Differential scanning calorimetry profile of nanocoating. The lines are shifted vertically for clarity. The glass transitions are also labelled. b) Profiles of thermal gravimetric analysis of nanocoatings. The coatings are thermally stable at 150 °C, the processing temperature.

Table S1. Summarized characteristics of the two sets of substrates used in the two case studies, G(TFET-*r*-2MP)$_{\varphi_2}$ and G(TFET-*r*-MTG)$_{\varphi_2}$.

Sample ID	φ_2	M_n (kg mol^{-1})	*Đ*	*N*[a]	R_g (nm)[b]
G(TFET-*r*-2MP)$_{\varphi_2}$	0	17.0	1.07	196.0	3.7
	0.213	16.9	1.06	195.2	3.7
	0.385	16.9	1.06	194.6	3.7
	0.536	16.8	1.08	194.0	3.7
	0.585	16.8	1.07	193.8	3.7
	0.617	16.8	1.04	193.7	3.7
	0.673	16.8	1.06	193.5	3.7
	1.000	16.7	1.06	192.3	3.7
G(TFET-*r*-MTG)$_{\varphi_2}$	0	17.0	1.07	196.0	3.7
	0.158	16.9	1.06	194.8	3.7
	0.215	16.9	1.09	194.3	3.7
	0.298	16.8	1.08	193.7	3.7
	0.520	16.7	1.08	192.0	3.7
	0.769	16.5	1.09	190.1	3.7
	0.853	16.4	1.09	189.5	3.7
	1.000	16.3	1.08	188.4	3.6

[a] The degree of polymerization, *N*, is estimated using the equation, $N = \frac{1}{144}\frac{1}{N_A}\frac{M_n}{\rho}$, where ρ is the density of the polymer (ρ = 1.00 g cm^{-3} is used in this work) and N_A is the Avogadro's number (N_A = 6.022 × 10^{23} mol^{-1} is used). The equation assumes a reference volume of 144 Å^3.

[b] Radius of gyration, R_g, is estimated using equation (3) in the main text.
